# IHPP: Improved Human Parts as Points for Multi-Person Pose Regression

**DOI:** 10.3390/s26102916

**Published:** 2026-05-07

**Authors:** Hao Xu, Yihan Liu, Yuting Fan, Shuyue Zhou, Kenan Lou, Xingfa Shen, Yabo Xiao

**Affiliations:** 1 Computer Science and Technology, Lishui University, Lishui 323000, China; 20180001@lsu.edu.cn (H.X.); eateateatcookies@gmail.com (Y.L.); gnit_fyt@126.com (Y.F.); studyzsy@126.com (S.Z.); 2 Jinyun County Lishui University Jinyun Industrial Innovation Research Institute, Lishui University, Lishui 323000, China; 3 Computer Science and Technology, Hangzhou Dianzi University, Hangzhou 310018, China; 4 School of Electronic Engineering, Beijing University of Posts and Telecommunications, Beijing 100876, China; xiaoyabo@bupt.edu.cn

**Keywords:** human pose estimation, body parts, semantic proxy points, long-range extremity, group-wise convolution, efficiency–accuracy tradeoff

## Abstract

Multi-personpose estimation is a fundamental technology for deep learning-based intelligent sensing systems, enabling downstream understanding of human action in applications such as surveillance, robotics, healthcare, and sports analytics. Most two-stage multi-person pose estimation algorithms suffer from low efficiency due to their decoupled representation between body and keypoints, resulting in a complex inference process. Single-stage algorithms such as AdaptivePose that represent body parts as semantic proxy points can substantially simplify the pipeline while achieving competitive results; however, the limited receptive field of convolutional features still makes it difficult to localize proxy points for long-range extremities in semantically informative regions. IHPP addresses this issue as a task-specific refinement of AdaptivePose; it enriches the proxy-point perceiver with directional context modeling along the horizontal and vertical axes and redesigns the second-step regression stage with a part-wise branch to reduce inter-part feature interference while keeping the overall pipeline lightweight. Here, we present IHPP in three sizes (IHPP-S, IHPP-M, and IHPP-L), optimizing the tradeoffs between efficiency and accuracy through fine-tuning of the channel dimensions for each bodypart feature. IHPP-L achieves 72.3 AP at 28 fps on COCO test-dev, surpassing DEKR-W48 and SWAHR-W48 by 1.3 AP and 0.3 AP, respectively, while IHPP-M reaches 69.0 AP at 35 fps and IHPP-S runs at 42 fps with only 9.4 M parameters. On CrowdPose, IHPP-M outperforms AdaptivePose-W48 by 0.2 AP with about one-third the parameters. Comprehensive experiments on the MS COCO and CrowdPose datasets validate the effectiveness of this design.

## 1. Introduction

Human pose estimation has emerged as a core component of deep learning-based perception for intelligent sensor systems, where visual information captured by camera sensors needs to be transformed into structured representations of human motion. Precise multi-person pose estimation not only enhances human-centric applications such as intelligent surveillance, human–robot interaction, and sports analytics but also serves as a critical foundation for higher-level tasks, including human action recognition and behavior analysis in complex environments. Despite significant advances in deep learning over recent years, multi-person scenarios remain challenging due to factors such as occlusion, inter-person interactions, and substantial variations in scale and crowd density, which complicate the simultaneous achievement of high accuracy and real-time efficiency.

The primary objective of the multi-person pose estimation task is to predict the keypoint locations of all individuals in an image. This plays a pivotal role in human-centric perception, generation, and diverse applications. Existing methods for multi-person pose estimation can be summarized into two-stage and one-stage pipelines.

Two-stage pipelines are further divided into top-down and bottom-up approaches. Top-down approaches [1,2,3,4,5,6,7,8] first employ a detection model to predict human body bounding boxes for localizing individuals, followed by individual pose estimation within detected regions. However, the efficiency of this approach is limited by the two-stage process and the number of individuals in the image. Bottom-up approaches [9,10,11,12,13,14,15] employ a single model to predict keypoints positions and their associations from the entire image, followed by grouping keypoints through complex postprocessing heuristics. Both approaches face major challenges in balancing prediction accuracy and inference speed; furthermore, heavy pipelines often require extensive engineering tuning, hindering models’ representational capacity and efficiency.

One-stage pipelines directly predict instance-aware keypoints using either query-based or center-based approaches. Query-based approaches [16,17,18] leverage the modeling capacity of transformers and employ complex encoders or decoders to regress multi-instance keypoints; however, their high computational cost limits practical deployment. On the other hand, center-based approaches [19,20,21,22,23] represent instances by their center pixels and predict the corresponding keypoints through displacements or instance-aware heatmaps. Compared with query-based approaches, center-based approaches use lighter models and achieve higher computational efficiency.

AdaptivePose [22] presents compact pose representation through a center-based approach, characterizing bodyparts with adaptive proxy points before final keypoint regression. However, due to the limited receptive field of convolutional features it remains difficult to effectively capture proxy points of long-range extremities, which are prone to drift toward semantically weak regions such as the background. As a result, the second-step regression is guided by less informative features, which weakens pose regression under challenging spatial layouts.

**Figure 1:**
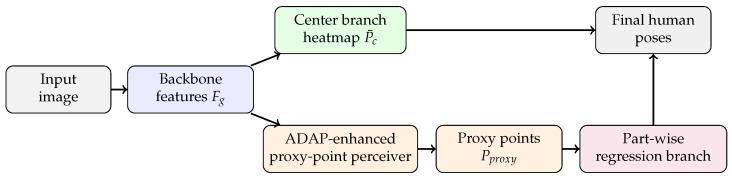
High-level pipeline of IHPP. A shared backbone generates features for center localization and the ADAP proxy-point perceiver. The resulting proxy-point features are processed by the part-wise regression branch to predict final keypoints.

To address this issue, we propose IHPP, an efficient proxy-point-based framework that refines AdaptivePose at two task-specific locations. As illustrated in Figure 1, IHPP predicts person centers and bodypart proxy points in parallel from a shared backbone feature map. First, we replace the local proxy-point perceiver with ADAP, meaning that the first-step offsets are predicted from a feature map enriched by directional context along the horizontal and vertical axes. Second, we replace the original shared second-step regressor with a part-wise regression branch that processes different kinematic parts with reduced feature competition. This design directly targets the instability of proxy-point localization for long-range extremities while preserving the efficiency of the overall single-stage pipeline.

Our key contributions are summarized as follows:

- A task-specific ADAP module that injects directional contextual reasoning into the proxy-point perceiver to improve the localization of long-range extremity proxy points within the AdaptivePose-style regression pipeline.
- A part-wise regression branch that reduces feature interference across bodyparts while lowering the parameter cost of the second-step keypoint regression stage.
- A scalable IHPP family with small, medium, and large variants, offering different efficiency–accuracy tradeoffs for practical sensing scenarios.

## 2. Related Work

We briefly review three lines of work that are relevant to this study: two-stage multi-person pose estimation, one-stage regression-based pose estimation, and axis-decoupled context modeling. This organization clarifies the methodological landscape surrounding efficient multi-person pose estimation and long-range context modeling.

### 2.1. Two-Stage Methods

The two-stage paradigm dominates current mainstream methods for multi-person pose estimators. It can be further classified into top-down methods and bottom-up methods that divide the task into two sequential sub-tasks. Top-down methods [1,7,8,24,25] disentangle the task into human detection and single-person pose estimation, mainly focusing on keypoint representation learning and improving the accuracy of single-person pose estimation; however, they suffer from heavy reliance on the performance of the human detector, redundant computational costs for additional human detection and RoI operations, and separate training for the human detector and corresponding pose estimator. Bottom-up methods [9,13,14,15] sequentially conduct human-agnostic keypoint detection and instance grouping; however, the grouping process is hand-crafted and time-consuming, sacrificing the model’s representation capacity and efficiency.

### 2.2. One-Stage Methods

Another line is the one-stage paradigm, including both query-based methods and center-based methods that directly predict instance-aware keypoints. Query-based methods [16,17,18] rely on the strong modeling capacity of transformers and use complex encoders or decoders to regress multi-instance keypoints. However, their high computational cost limits practical deployment. Center-based methods [19,20,21,22,23] represent each instance by its center pixel and predict the corresponding keypoints through displacements or instance-aware heatmaps. Compared with query-based methods, center-based methods generally use lighter models and achieve higher computational efficiency. AdaptivePose [22] further introduces semantic proxy points as intermediate nodes for two-step offset regression, illustrating the promise of proxy point-based center regressors. IHPP follows this proxy point-based regression line rather than proposing a new pose representation, retaining the AdaptivePose-style two-step regression pipeline but strengthens the proxy-point perceiver with task-specific axis-decoupled context modeling and redesigning the second-step regressor with a part-wise branch. Nevertheless, long-range context modeling for extremity-related proxy points remains less explored in this family of methods.

### 2.3. Axial and Efficient Context Modeling

Recent studies have shown that factorizing 2D attention into 1D operations along spatial axes can substantially enlarge the receptive field while reducing the cost of dense self-attention [26,27]. In particular, Axial Attention [26] and Axial-DeepLab [27] use sequential horizontal and vertical attention as a general context modeling mechanism for multidimensional transformers or dense prediction backbones. Likewise, related efficient formulations exploit directional decomposition to approximate long-range 2D interactions with lower cost than dense attention. IHPP is closely related to this line of work in its use of axis-decoupled context aggregation, but differs in its scope and objective. We do not claim a new axial-attention family, and ADAP is not used as a general-purpose backbone replacement; instead, we adapt this principle to the proxy-point perceiver of a lightweight pose regression framework in which the goal is to stabilize the first-step localization of extremity-related proxy points while preserving the efficiency of the overall pipeline.

## 3. Methods

### 3.1. Preliminaries

AdaptivePose [22] proposes an expressive pose representation method in a pixel-wise pose regression framework, with human parts termed as adaptive points. In this manner, the human pose is divided into several kinematic parts and each kinematic part is represented by a semantic proxy point. Instead of directly regressing keypoints using a center feature with limited representation capacity, AdaptivePose utilizes bodypart proxy points as intermediate nodes to perform keypoint regression in a two-step process. The first step involves a bodypart perceiver to regress bodypart proxy points using the features from the central point. The second step uses the features of these local proxy points to regress the keypoint offsets within the corresponding area. Note that the local semantic proxy points are not under explicit supervision; supervision is applied only to the cumulative sum of the two-step offsets. The gradients help the proxy points to locate semantically rich locations, enabling accurate prediction of the second-step offset. This representation effectively and efficiently models the associations between human keypoints and corresponding individuals while precisely regressing the positions of multi-person keypoints.

The pixel-wise regression framework utilizes a convolutional network to generate visual feature $F_{g}$, followed by two parallel branches, namely, the center localization branch and keypoint regression branch. The keypoint regression branch adopts two-step offset regression.

**Figure 2:**
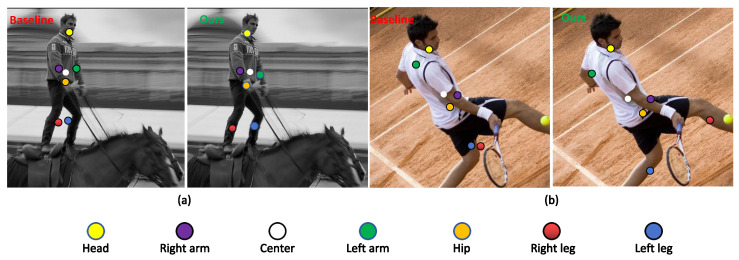
Qualitative comparison of proxy-point localization between AdaptivePose and IHPP. In each subfigure, the left image shows AdaptivePose (Baseline) and the right image shows IHPP (Ours). (**a**) Horse-riding example, where AdaptivePose places several long-range leg proxy points in background regions, whereas IHPP localizes them closer to the corresponding foreground body-part regions. (**b**) Tennis-player example, where IHPP produces proxy points that are more consistent with the visible body-part regions than AdaptivePose. The result illustrates the effect of ADAP, where standard self-attention is applied along the decoupled horizontal and vertical axes for proxy-point perception.

Intuitively, when proxy points are located in semantically informative regions, the corresponding features can better encode information about the related bodyparts and support more accurate second-step offset regression. However, for long-range extremities the learned proxy points are often localized in background regions, which limits the expressive capacity of the model, as shown in Figure 2.

These observations motivate two design goals for IHPP: enriching the proxy-point perceiver with stronger long-range context, and reducing feature interference in the second-step regression. Accordingly, Section 3.2 introduces the Axis-Decoupled Attention Perceiver (ADAP), which first decomposes the 2D proxy-point feature into horizontal and vertical axes and then applies standard self-attention along each axis to guide extremity-related proxy points towards semantically rich regions. Section 3.3 presents the part-wise regression branch used to decouple the regression of different kinematic parts.

From a modeling perspective, IHPP introduces two task-specific modifications, rather than a new pose representation or a new generic attention operator. First, we replace the local bodypart perceiver with ADAP to perform axis-wise contextual aggregation before the first-step offset regression. In this design, “axis-decoupled” refers to the spatial factorization of the proxy-point feature, while the attention operator used on each axis is standard self-attention. Second, we redesign the second-step regression head as a part-wise branch so that different kinematic parts can be processed with less feature competition. These two components are described in Section 3.2 and Section 3.3, respectively.

This clarification is important because the novelty of IHPP does not lie in proposing entirely new generic building blocks in isolation; instead, it lies in adapting these known design principles to the specific failure mode of proxy point-based pose regression, that is, its unstable localization of long-range extremity proxy points, as well as in showing that the resulting part-wise design incorporating ADAP yields complementary gains in both accuracy and efficiency.

### 3.2. Axis-Decoupled Attention Perceiver

**Figure 3:**
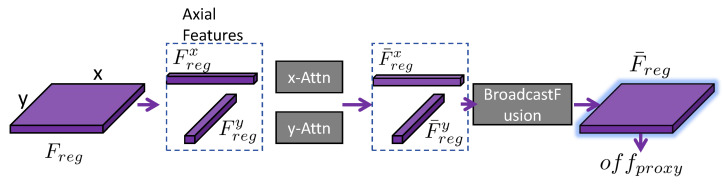
Structure of the Axis-Decoupled Attention Perceiver (ADAP).

AdaptivePose employs a standard 3 × 3 convolutional layer as the part perceiver to predict the positions of bodypart proxy points. However, due to the limited receptive field of convolutional features, it is difficult to drive the proxy points of long-range extremities towards semantically informative regions. The structure of the proposed ADAP module is illustrated in Figure 3.

ADAP shares the same high-level intuition as prior axial attention designs, namely, that a 2D interaction can be approximated efficiently by sequential 1D context aggregation along the horizontal and vertical axes [26,27]. Therefore, we position ADAP as a task-specific adaptation of this existing design principle rather than as a new general-purpose attention family. In IHPP, ADAP is used only for proxy-point perception, where the goal is to stabilize the first-step localization of semantic proxy points for long-range extremities under the efficiency constraints of a lightweight single-stage pipeline.

Formally, let $F_{reg}\in R^{C\times H\times W}$ denote the regression feature map used to predict the first-step offsets. We first aggregate it along the two spatial axes to obtain directional descriptors

(1) $$F_{reg}^{x}\left(c,1,w\right)=\frac{1}{H}\sum_{h=1}^{H}F_{reg}\left(c,h,w\right),\;F_{reg}^{y}\left(c,h,1\right)=\frac{1}{W}\sum_{w=1}^{W}F_{reg}\left(c,h,w\right),$$

where $F_{reg}^{x}\in R^{C\times 1\times W}$ summarizes horizontal context and $F_{reg}^{y}\in R^{C\times H\times 1}$ summarizes vertical context. For each axis a∈{x,y}, we apply learnable 1×1 projections $W_{q}^{a}$, $W_{k}^{a}$, $W_{v}^{a}$, and $W_{o}^{a}$ to obtain

(2) $$Q^{a}=W_{q}^{a}F_{reg}^{a},\;K^{a}=W_{k}^{a}F_{reg}^{a},\;V^{a}=W_{v}^{a}F_{reg}^{a},$$

where $Q^{x},K^{x},V^{x}\in R^{d\times W}$ and $Q^{y},K^{y},V^{y}\in R^{d\times H}$. The corresponding axis-wise attention matrices are

(3) $$A^{x}=\mathrm{Softmax}\;\left(\frac{{\left(Q^{x}\right)}^{⊤}K^{x}}{\sqrt{d}}\right)\in R^{W\times W},\;A^{y}=\mathrm{Softmax}\;\left(\frac{{\left(Q^{y}\right)}^{⊤}K^{y}}{\sqrt{d}}\right)\in R^{H\times H}.$$

The contextualized axis features are then written as

(4) $${\hat{F}}_{reg}^{x}=W_{o}^{x}\left(V^{x}A^{x⊤}\right)\in R^{C\times 1\times W},\;{\hat{F}}_{reg}^{y}=W_{o}^{y}\left(V^{y}A^{y⊤}\right)\in R^{C\times H\times 1}.$$

Finally, we broadcast the two directional responses back to the 2D grid and fuse them with the original regression feature map:

(5) $${\bar{F}}_{reg}\left(c,h,w\right)=F_{reg}\left(c,h,w\right)+{\hat{F}}_{reg}^{x}\left(c,1,w\right)+{\hat{F}}_{reg}^{y}\left(c,h,1\right).$$

The resulting ${\bar{F}}_{reg}\in R^{C\times H\times W}$ is passed to a prediction head $g_{proxy}$, implemented as a lightweight convolutional regressor, to estimate the first-step offsets

(6) $$off_{proxy}=g_{proxy}\left({\bar{F}}_{reg}\right),\;off_{proxy}\in R^{2K\times H\times W},$$

where *K* is the number of semantic proxy points. In this formulation, the learnable parameters of ADAP are exactly the projection matrices ${\left\{W_{q}^{a},W_{k}^{a},W_{v}^{a},W_{o}^{a}\right\}}_{a\in \{x,y\}}$ together with the output head $g_{proxy}$.

### 3.3. Part-Wise Regression Branch

**Figure 4:**
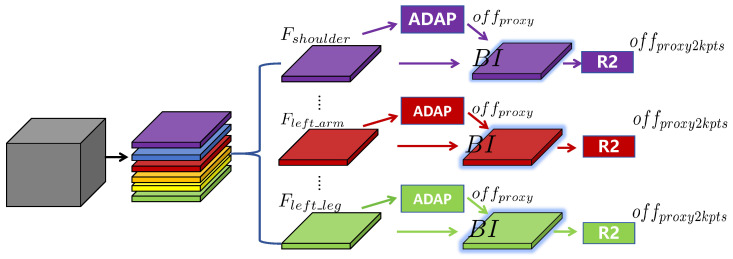
Detailed structure of the part-wise regression branch in IHPP. BI indicates bilinear interpolation.

Based on the estimated proxy points $P_{proxy}$, we use the regressor $R_{2}$ to predict the corresponding keypoints. The process can be denoted as $F_{proxy}=Bilinear\_inp\left(F_{k},P_{proxy}\right)$, $off_{proxy2kpts}=R_{2}\left(F_{proxy}\right)$, where $F_{k}$ is used to extract the features of proxy points via bilinear interpolation. As shown in Figure 4, we redesign the second-step regression head so that different kinematic parts can be processed with reduced feature competition. Specifically, we introduce the group-wise concept from convolution into the macro regression branch and partition the feature into *K* groups, namely, $F_{head-shoulder}$, $F_{left\_arm}$, $F_{right\_arm}$, $F_{hip}$, $F_{left\_leg}$, and $F_{right\_leg}$, corresponding to six distinct bodyparts. For instance, the part-wise feature $F_{hip}$ is fed into a dedicated regressor $R_{2}^{hip}$ to locate the keypoints in the hip part. The part-wise design explicitly facilitates feature learning for different bodyparts while reducing the parameter burden of the regression branch. We also observe that the channel dimensions of part features need to be compatible with the scale of the backbone in order to fully exploit the capacity of different IHPP variants.

### 3.4. Training and Inference

IHPP follows a pixel-wise regression framework and adopts the DLA [28] as the backbone to extract general features. The center localization branch predicts a center heatmap ${\bar{P}}_{c}$ to indicate the confidence at each pixel. We utilize the pixel-wise focal loss [29] in a penalty-reduced fashion to supervise the center heatmap:

(7) $${loss}_{hm}=\frac{1}{N}\sum_{n=1}^{N}\left\{\begin{array}{ccc} {\left(1-{\bar{P}}_{c}\right)}^{\alpha }\;\ln\left({\bar{P}}_{c}\right) & \; & ifP_{c}=1,\\ {\left(1-P_{c}\right)}^{\beta }\;{\bar{P}}_{c}^{\alpha }\;\ln\left(1-{\bar{P}}_{c}\right) & \; & elifP_{c}\neq 1, \end{array}\right.$$

where N indicates the number of positives, $P_{c}$ refers to ground truth, and α, β are hyperparameters set to 2 and 4, respectively, to balance positives/negatives. In parallel, we employ the vanilla L1 loss and object keypoint similarity loss to constrain cumulations of two-step offsets:

(8) $$Loss_{reg}=Loss_{oks}+Loss_{l1},$$

(9) $$Loss_{oks}=1-\frac{\sum_{i}exp\left(\frac{-d_{i}^{2}}{2s^{2}k_{i}^{2}}\right)\delta \left(\upsilon_{i}>0\right)}{\sum_{i}\delta \left(\upsilon_{i}>0\right)}.$$

The loss weights are set to 1, 5, and 1 for ${loss}_{hm}$, $Loss_{oks}$, and $Loss_{l1}$, respectively.

In the inference phase, we apply a 5 × 5 max-pooling kernel on the predicted center heatmap to identify the human center, maintaining 20 potential pose candidates. Meanwhile, we extract the associated offsets from the output of the part-wise regression branch to construct the human pose.

## 4. Experiments

### 4.1. Setup

**Dataset**

We evaluate IHPP on two widely-used 2D MPPE datasets, MS COCO [30] and CrowdPose [5]. MS COCO is a large-scale benchmark consisting of more than 250k human instances annotated with 17 keypoints. We train our model on the COCO train2017 dataset with 57k images and evaluate it on both the COCO mini-val set with 5000 images and the test-dev2017 set with 20k images. The CrowdPose dataset, which integrates 20k images with annotations for 80k individuals, is partitioned into training, validation, and test subsets in a ratio of 5:1:4, offering a robust benchmark for assessing model performance in crowded scenarios.

**Evaluation Metrics**

For both the MS COCO and CrowdPose datasets, keypoint detection performance is quantified by average precision and average recall based on varying thresholds of the object keypoint similarity (OKS) metric [30].

**Data Augmentation**

During the training phase, we conduct random flipping (0.5), random rotation (−30, 30), and color jitter to enhance the training samples. Each input is cropped according to a random center and random scale, then resized to 512×512 for IHPP-S and IHPP-M or 640×640 for IHPP-L to obtain different speed–accuracy tradeoffs.

**Implementation Details**

We train all models with Adam [31] on a workstation with four RTX 3090 GPUs, and implement the framework in PyTorch 1.8.

Following the CenterNet training setup, the initial learning rate is $2.5\times 10^{-4}$, which is decayed by 10× at epoch 230; the total training length is 280 epochs, and the weight decay is 0.0. The total batch size is 128 for IHPP-S and IHPP-M and 64 for IHPP-L. We adopt DLA34-BottleNeck, DLA34-Basicblock, and DLA102 [28] for IHPP-S (9.4 M), IHPP-M (20.6 M), and IHPP-L (48.8 M), respectively. During training, IHPP-S and IHPP-M use 512×512 inputs, whereas IHPP-L uses 640×640 inputs. For IHPP efficiency evaluation, runtime is measured with PyTorch inference on a single RTX 3090 GPU at batch size 1 and reported as the average throughput over 5000 validation images, using a long-side resize of 512 for IHPP-S and IHPP-M and 640 for IHPP-L.

### 4.2. Ablation Experiments

We adopt AdaptivePose-DLA34 (21.0M) and IHPP-M (20.6M) to conduct ablation studies, and report the results on the COCO mini-val set without flipping or multi-scale testing to verify the effectiveness of the proposed ASAP and part-wise regression branch design.

As shown in Table 1, the AdaptivePose baseline adopts a vanilla 3 × 3 convolutional layer to perceive bodypart information, achieving 67.0 AP. Replacing the vanilla perceiver with a single ADAP block yields a 0.9 AP improvement, while stacking two ADAP blocks does not provide additional gains. PWR is motivated by the group-wise design principle in convolution: by partitioning the second-step regression branch into bodypart-specific groups, it reduces the parameter cost of the regression head from 1.2M to 0.6M. When used alone, this lightweight design causes only a marginal AP change (66.9 AP versus 67.0 AP), suggesting that most of the baseline performance is preserved while the regression branch becomes more parameter-efficient. When combined with ADAP, the model achieves the best result of 68.7 AP with only 0.8M regression-branch parameters, indicating that ADAP provides the main accuracy gain while PWR serves as a lightweight complementary design that maintains performance at a lower regression cost.

We investigate the different bodypart perceivers in Table 2. Compared with using deformable convolution [32] to capture regional semantics, the contextual modeling variants in ADAP obtain consistently better results. The similar AP values among ADAP-GAU, ADAP-EA, and ADAP-SA suggest that the main benefit comes from task-oriented contextual refinement in the proxy-point perceiver rather than from any claim that one attention formulation is intrinsically superior to another.

We further validate the channel settings for all model sizes in Table 3. For IHPP-S, the AP improves from 66.9 to 67.4 when increasing the per-part channel dimension from 32 to 48, while no additional gain is observed at 64 channels. Therefore, we choose 48 for IHPP-S to preserve the efficiency of the compact model. For IHPP-M, the performance improves as the channel dimension increases, then saturates around 64 channels, which supports using 64 as the reference setting for the medium-size model. For IHPP-L, increasing the per-part channel dimension from 96 to 128 improves AP from 68.9 to 69.6, indicating that the larger backbone benefits from a wider part-wise regression branch. Based on these results, we set the per-part channel dimensions to 48, 64, and 128 for IHPP-S, IHPP-M, and IHPP-L, respectively. This choice follows a monotonic scaling pattern and is supported by additional validation across all three model sizes.

### 4.3. Comprehensive Results

**MS COCO**

**Figure 5:**
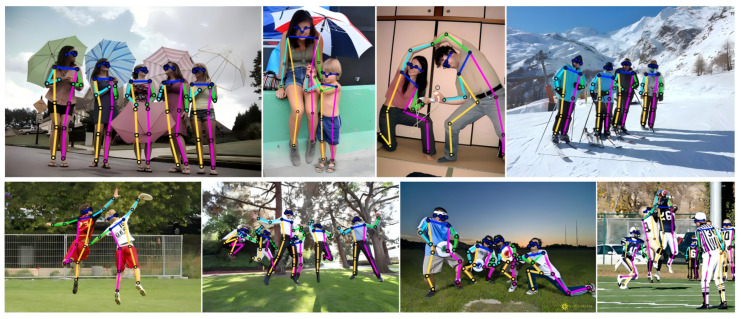
Pose regression results in various scenes. Colored dots denote predicted keypoints, and colored lines denote skeletal connections.

We list comprehensive comparisons in Table 4 and summarize representative efficiency evidence in Table 5. For bottom-up methods, IHPP-L (48.8 M) exceeds DEKR-W48 (65.7 M) and SWAHR-W48 (63.8 M) by 1.3 AP and 0.3 AP, respectively. For single-stage regression-based methods, IHPP-L achieves competitive accuracy with only about one quarter of the parameters when compared to the query-based EDPose and QueryPose methods. Under PyTorch inference on a single RTX 3090 GPU with batch size 1, IHPP-S, IHPP-M, and IHPP-L run at 42, 35, and 28 fps, respectively; these values are averaged over 5000 validation images using a long-side resize of 512 for IHPP-S and IHPP-M and 640 for IHPP-L. Table 5 reports these unified IHPP measurements together with representative competitor time (s), with values retained from prior reports as reference rather than same-hardware comparisons. IHPP-S achieves 66.3 AP and outperforms KAPAO-S and YoloPose-S with few parameters, while IHPP consistently achieves better results than KAPAO and YoloPose in other sizes. Moreover, we show some qualitative results for various complex scenes (e.g., occlusions and abnormal poses) in Figure 5.

**CrowdPose**

As shown in Table 6, we further verify the effectiveness of IHPP on CrowdPose. IHPP-S achieves 66.1 AP with only 9.4M parameters. IHPP-M surpasses AdaptivePose-W48, DEKR-W48, and KAPAO-L over 0.2, 1.4, and 0.5 AP with only one-third the parameters. Furthermore, our IHPP-L achieves competitive performance compared to transformer-based methods.

### 4.4. Quantitative Error Analysis

To provide a quantitative analysis of the remaining failure modes, we further compare IHPP-M with AdaptivePose-DLA34 on COCO mini-val subsets stratified by person scale and occlusion level. For scale analysis, we report AP on small, medium, and large person subsets in Table 7. For occlusion analysis, we compute the visibility ratio of each person as the number of visible keypoints divided by the number of annotated keypoints, where visible keypoints have v=2 and annotated keypoints have v>0. Subsets with low, medium, and heavy occlusion are defined using visibility ratio ≥0.75, 0.50≤ visibility ratio <0.75, and visibility ratio <0.50, respectively.

The scale-stratified results show that IHPP-M consistently improves over AdaptivePose-DLA34 across all person scales while using slightly fewer parameters. The gains are more evident on medium and large persons, indicating that IHPP can better model long-range extremities in semantically informative regions when sufficient visual evidence is available. The smaller gain on small persons suggests that extremely small instances remain challenging because of their proxy-point features being less spatially distinguishable.

The occlusion-stratified results in Table 8 show that IHPP-M also improves over AdaptivePose-DLA34 under all occlusion levels. The gain becomes larger as the occlusion level increases, indicating that ADAP’s long-range contextual modeling and the part-wise regression branch are helpful for occluded bodyparts and limb confusion. Nevertheless, the absolute AP under heavy occlusion remains much lower than that under low occlusion, suggesting that severe mutual occlusion and overlapping limbs remain important failure modes.

## 5. Limitations and Future Work

Despite the promising results, the current study still has several limitations. First, although the quantitative error analysis shows that IHPP improves over AdaptivePose across different person scales and occlusion levels, extremely small persons and heavily occluded instances still remain difficult to estimate accurately. Second, although we report efficiency results for different model variants, the speed evaluation could be further strengthened through more systematic comparisons under unified hardware and evaluation protocols. Third, the current channel-scaling strategy is heuristic and may not be optimal for all backbone sizes.

In future work, we plan to explore stronger scale-aware modeling for small-person pose estimation, conduct richer failure-case analysis under severe occlusion, and investigate more systematic architecture and efficiency optimizations.

## 6. Conclusions

We present IHPP, an efficient single-stage multi-person pose estimator built upon the proxy-point representation of AdaptivePose. IHPP improves proxy point-based pose regression through two task-specific design refinements: ADAP for context-enriched proxy-point perception, and a part-wise regression branch for second-step offset prediction. Together, these designs improve the localization of long-range extremities while reducing inter-part feature interference and the parameter cost of the regression stage. We further instantiate IHPP in three model variants to provide flexible efficiency–accuracy tradeoffs and demonstrate the effectiveness of this design on both COCO and CrowdPose.

## Figures and Tables

**Table 1:** Ablation studies: Baseline denotes AdaptivePose-DLA34; ADAP-n is the Axis-Decoupled Attention Perceiver (ADAP), where n denotes the number of stacked ADAP blocks; PWR indicates part-wise regression; Params. indicates the parameters of keypoint regression branch. A checkmark indicates that the corresponding module is used, whereas a hyphen indicates that it is not used.

ADAP-1	ADAP-2	PWR	Params.	AP	${\mathrm{AP}}_{M}$	${\mathrm{AP}}_{L}$
-	-	-	1.2 M	67.0	61.2	76.0
√	-	-	1.9 M	67.9	62.6	76.7
-	√	-	2.5 M	67.8	62.1	76.8
-	-	√	0.6 M	66.9	61.4	75.9
√	-	√	0.8 M	68.7	63.1	77.3

**Table 2:** Comparison with other bodypart semantic perceivers, including DCN [32], ADAP-GAU (Gated Attention Unit [33]), ADAP-EA (Efficient Attention [34]), and ADAP-SA (Self-Attention [35]).

Perceiver	AP	${\mathrm{AP}}_{M}$	${\mathrm{AP}}_{L}$
Part-wise DCN	67.7	62.2	76.4
Part-wise ADAP-GAU	68.4	62.6	76.9
Part-wise ADAP-EA	68.6	62.9	77.4
Part-wise ADAP-SA	68.7	63.1	77.3

**Table 3:** Results on varying per-part channel dimensions in the part-wise regression branch across IHPP-S, IHPP-M, and IHPP-L. A hyphen indicates that the corresponding channel setting was not evaluated.

Channel	32	48	64	96	128
Network					
IHPP-S	66.9	67.4	67.4	-	-
IHPP-M	68.1	68.5	68.7	68.6	68.7
IHPP-L	-	-	-	68.9	69.6

**Table 4:** Comparison with state-of-the-art methods on the COCO test-dev dataset; * indicates the use of extra test-time refinements. A hyphen indicates that the number of parameters is not reported.

Methods	Params. (M)	AP	${\mathrm{AR}}_{50}$	${\mathrm{AR}}_{75}$	${\mathrm{AP}}_{M}$	${\mathrm{AP}}_{L}$
**Bottom-Up Methods**						
CMU-Pose * [9]	-	61.8	84.9	67.5	57.1	68.2
AE * [14]	227.8	65.5	86.8	72.3	60.6	72.6
CenterNet-HG [29]	-	63.0	86.8	69.6	58.9	70.4
HrHRNet-W48 * [15]	63.8	70.5	89.3	77.2	66.6	75.8
FCPose-R101 [23]	-	65.6	87.9	72.6	62.1	72.3
SWAHR-W48 * [13]	63.8	72.0	90.7	78.8	67.8	77.7
DEKR-W48 * [12]	65.7	71.0	88.3	77.4	66.7	78.5
**Single-Stage Methods**						
KAPAO-S [36]	12.6	63.8	88.4	70.4	58.6	71.7
YoloPose-S [37]	10.8	63.2	87.8	69.5	57.6	72.6
**IHPP-S**	**9.4**	66.3	88.7	73.1	62.7	74.5
KAPAO-M [36]	35.8	68.8	90.5	76.5	64.3	76.0
YoloPose-M [37]	29.3	68.6	90.7	75.8	63.4	77.1
**IHPP-M**	**20.6**	69.0	90.2	76.1	63.7	75.7
QueryPose (Swin-L) [16]	-	72.2	92.0	78.8	67.3	79.4
EDPose (Swin-L) [17]	218.0	72.7	92.3	80.9	67.6	80.0
PETR (Swin-L) [18]	213.8	70.5	91.5	78.7	65.2	78.0
KAPAO-L [36]	77.0	70.3	91.2	77.8	66.3	76.8
AdaptivePose-w48 [22]	64.7	71.4	90.2	78.5	66.8	78.2
YoloPose-L [37]	61.3	70.2	91.1	77.8	65.3	78.2
**IHPP-L**	**48.8**	72.3	92.3	80.1	66.8	79.4

**Table 5:** Efficiency comparison on COCO using representative competitor timings from prior reports and unified IHPP measurements. Time(s) denotes the per-image inference time. IHPP speeds are measured with PyTorch inference on a single RTX 3090 GPU at batch size 1 and averaged over 5000 validation images using a long-side resize of 512 for IHPP-S and IHPP-M and 640 for IHPP-L; competitor timings are retained as literature reference values rather than same-hardware measurements.

Methods	Params. (M)	AP	Long Side	Time (s)	FPS
**Representative Competitors (reference timings)**					
DEKR-W32 [12]	29.6	63.4	512	0.1450	6.9
HigherHRNet-W32 [15]	28.6	63.6	512	0.1640	6.1
SWAHR-W32 [13]	28.6	64.7	512	0.2350	4.3
AdaptivePose (DLA-34) [22]	21.0	65.5	512	0.0340	29.4
DEKR-W48 [12]	65.7	67.1	640	0.1950	5.1
HigherHRNet-W48 [15]	63.8	66.6	640	0.2420	4.1
SWAHR-W48 [13]	63.8	67.3	640	0.4280	2.3
**IHPP (unified RTX 3090 measurements)**					
IHPP-S	9.4	66.3	512	0.0238	42.0
IHPP-M	20.6	69.0	512	0.0286	35.0
IHPP-L	48.8	72.3	640	0.0357	28.0

**Table 6:** Comparison with the state-of-the-art methods on the CrowdPose test set. ^†^ indicates results obtained with multi-scale testing.

Methods	AP	${\mathrm{AP}}_{50}$	${\mathrm{AP}}_{75}$	${\mathrm{AP}}_{E}$	${\mathrm{AP}}_{M}$	${\mathrm{AP}}_{H}$
Rmpe [8]	61.0	81.3	66.0	71.2	61.4	51.1
SimpleBaseline [3]	60.8	84.2	71.5	71.4	61.2	51.2
CrowdPose [5]	66.0	84.2	71.5	75.5	66.3	57.4
HigherHRNet-W48 [15]	67.6	87.4	72.6	75.8	68.1	58.9
CenterGroup [38]	67.6	87.7	72.7	73.9	68.2	60.3
DEKR-W32 ^†^ [12]	67.0	85.4	72.4	75.5	68.0	56.9
DEKR-W48 ^†^ [12]	68.0	85.5	73.4	76.6	68.8	58.4
AdaptivePose-W48 [22]	69.2	87.3	75.0	76.7	70.0	60.9
KAPAO-L [13]	68.9	89.4	75.6	76.6	69.9	59.5
PETR(swin-L) [18]	71.6	90.4	78.3	77.3	72.0	65.8
IHPP-S	66.1	85.4	71.3	74.1	66.6	56.9
IHPP-M	69.4	87.9	75.3	77.1	70.2	60.2
IHPP-L	72.9	89.9	79.9	80.1	72.8	64.2

**Table 7:** Scale-stratified AP comparison on COCO mini-val. A hyphen indicates that the parameter count is not applicable to the gain row.

Method	Params	Small AP	Medium AP	Large AP
AdaptivePose-DLA34	21.0M	51.4	61.2	76.0
IHPP-M	20.6M	52.0	63.1	77.3
Gain	-	+0.6	+1.9	+1.3

**Table 8:** Occlusion-stratified AP comparison on COCO mini-val. A hyphen indicates that the parameter count is not applicable to the gain row.

Method	Params	Low Occ. AP	Medium Occ. AP	Heavy Occ. AP
AdaptivePose-DLA34	21.0M	82.1	70.2	53.3
IHPP-M	20.6M	82.6	71.3	55.1
Gain	-	+0.5	+1.1	+1.8

## Data Availability

Publicly available datasets were analyzed in this study. These data can be found in the MS COCO and CrowdPose datasets. Additional results generated during this study are contained within the article.
